# Staphylococcal leukotoxins trigger free intracellular Ca^2+^ rise in neurones, signalling through acidic stores and activation of store-operated channels

**DOI:** 10.1111/cmi.12069

**Published:** 2012-12-06

**Authors:** Emmanuel Jover, Mira Y Tawk, Benoît-Joseph Laventie, Bernard Poulain, Gilles Prévost

**Affiliations:** 1 INCI – UPR-CNRS 3212, Neurotransmission et sécrétion neuroendocrine5, rue Blaise Pascal, F- 67084, Strasbourg cedex, France; 2 Université de Strasbourg, Physiopathologie et Médecine Translationnelle EA-4438, Hôpitaux Universitaires de Strasbourg, Institut de Bactériologie3, rue Koeberlé, F-67000, Strasbourg, France; 3 Biozentrum; University of BaselKlingelbergstrasse 50/70, CH - 4056, Basel, Switzerland

## Abstract

Headache, muscle aches and chest pain of mild to medium intensity are among the most common clinical symptoms in moderate *Staphylococcus aureus* infections, with severe infections usually associated with worsening pain symptoms. These nociceptive responses of the body raise the question of how bacterial infection impinges on the nervous system. Does *S. aureus*, or its released virulence factors, act directly on neurones? To address this issue, we evaluated the potential effects on neurones of certain bi-component leukotoxins, which are virulent factors released by the bacterium. The activity of four different leukotoxins was verified by measuring the release of glutamate from rat cerebellar granular neurones. The bi-component γ-haemolysin HlgC/HlgB was the most potent leukotoxin, initiating transient rises in intracellular Ca^2+^ concentration in cerebellar neurones and in primary sensory neurones from dorsal root ganglia, as probed with the Fura-2 Ca^2+^ indicator dye. Using pharmacological antagonists of receptors and Ca^2+^ channels, the variations in intracellular Ca^2+^ concentration were found independent of the activation of voltage-operatedCa^2+^ channels or glutamate receptors. Drugs targeting Sarco-Endoplasmic Reticulum Ca^2+^-ATPase (SERCA) or H^+^-ATPase and antagonists of the store-operated Ca^2+^ entry complex blunted, or significantly reduced, the leukotoxin-induced elevation in intracellular Ca^2+^. Moreover, activation of the ADP-ribosyl cyclase CD38 was also required to initiate the release of Ca^2+^ from acidic stores. These findings suggest that, prior to forming a pore at the plasma membrane, leukotoxin HlgC/HlgB triggers a multistep process which initiates the release of Ca^2+^ from lysosomes, modifies the steady-state level of reticular Ca^2+^ stores and finally activates the Store-Operated Calcium Entry complex.

## Introduction

*Staphylococcus aureus* is a common host in human flora that asymptomatically colonizes one in three healthy individuals; children having higher persistent carriage rates than adults (Wertheim *et al*., 2005). However, this bacterium can be a serious pathogen, and is now the second cause of hospital and community-acquired infections worldwide (David and Daum, 2010; DeLeo *et al*., 2010; David *et al*., 2011). The severity and locations of *S. aureus* infections display broad variation. Contamination typically progresses from a local infection (furuncle, cellulitis, wound infection) to systemic propagation (bacteraemia) and metastatic infections (pneumonia, endocarditis, osteomyelitis, septic arthritis). The emergence and spread of community-acquired, drug-resistant (CA-MRSA) and extremely virulent strains reinforces the morbidity of *S. aureu*s infections (Chambers and DeLeo, 2009; David and Daum, 2010). Pathogenesis of the bacterium relies on an arsenal of virulence-associated factors, some of which specifically produce toxin-induced diseases (e.g. bullous impetigo, toxic shock syndrome, staphylococcal scaled skin syndrome and food-borne gastroenteritis) (Archer, 1998; Lowy, 1998).

One distinctive feature of CA-MRSA strains is the bearing of genes for the Panton-Valentine leukocidin (PVL), a bi-component toxin encoded by *lukS-PV* and *lukF-PV* from an integrated bacteriophage, rarely carried by *S. aureus* strains prior to the 1990s (Woodin, 1960; Finck-Barbançon *et al*., 1991; Rahman *et al*., 1992; Kaneko *et al*., 1998). The activity of PVL plays a role in pathogenesis under conditions involving host susceptibility factors and infected tissues (Gillet *et al*., 2002; Crémieux *et al*., 2009). Moreover, *S. aureus* has the capacity to produce several homologous two-component leukotoxins that contribute to the bacterium's power of infection, including the ubiquitous γ-haemolysins (Hlg, encoded by *hlgA*, *hlgB* and *hlgC*) (Prévost *et al*., 1995), the leukotoxin LukE–LukD (Gravet *et al*., 1998), PVL, LukM and LukF′-PV (Choorit *et al*., 1995). These toxins assemble as pore-forming octamers on the surface of susceptible target cells, such as neutrophils, monocytes and macrophages, and can thereby alter host cell functions or cause cytolysis (Prévost *et al*., 2001; Jayasinghe and Bayley, 2005). Genes encoding several cytolytic toxins, notably γ-haemolysin subunits HlgA, HlgB and HlgC, can be upregulated in human blood over time (Malachowa *et al*., 2011).

Major advances have been achieved over the last decade in the understanding of the structure and mechanisms of pore formation by two-component toxins from *S. aureus* and from other origins (Menestrina *et al*., 2003; Gonzalez *et al*., 2008). In contrast, much less is known regarding the overall cell targets and responses to *S. aureus* leukotoxins. The increase in free intracellular calcium or the loss of cellular potassium are examples of early cellular reactions to the presence of pore-forming toxins, an ion imbalance that may lead to the activation of a variety of signalling cascades (Woodin and Wieneke, 1963; Staali *et al*., 1998; Kloft *et al*., 2009; Kao *et al*., 2011).

In the present study, the question of the sensitivity of neurones to bi-component leukotoxins of *S. aureus* is addressed with the aim of deciphering the mechanisms of cell response. Cell cultures from rat cerebellar cortex and primary sensory neurones from dorsal root ganglia were used to demonstrate the neurotoxic activity of PVL, α-toxin and γ-haemolysins. The four virulence-associated factors maintained neuronal integrity while triggering a [Ca^2+^]_i_ increase in cerebellar neurones, which was followed by the release of glutamate. The γ-leukotoxin HlgC/HlgB was found to be the most potent on both types of cultured neurones. The leukotoxin-induced rises in free intracellular ([Ca^2+^]_i_) were initiated by a discharge of Ca^2+^ from acidic stores and followed by a Ca^2+^-induced Ca^2+^ release from the reticulum. These effects were further amplified by the activation of the store-operated Ca^2+^ entry complex. The initial signal linking leukotoxin binding to the acidic stores was due to the activation of the ADP ribosyl cyclase CD38. This cellular response to the presence of the leukotoxin in neuronal cells preceded the formation of toxin pores into the plasma membrane.

## Results

### Staphylococcal leukotoxins trigger glutamate release from cerebellar granular neurones

**Fig. 1:**
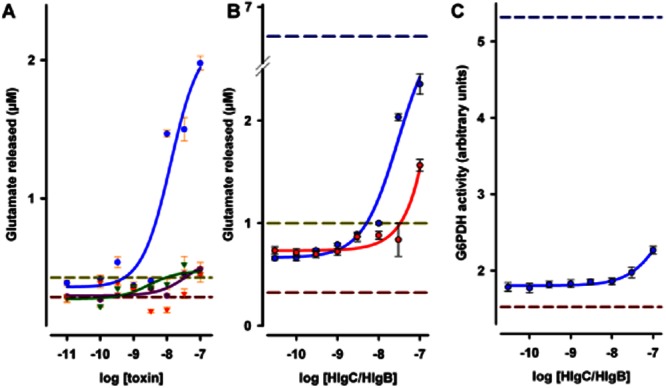
*Staphylococcus aureus* leukotoxins trigger glutamate release from cerebellar granular neurones. A. Cultured cells were challenged for 10 min with the indicated concentrations of leukotoxin HlgA/HlgB (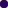), leukotoxin HlgC/HlgB (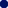), α-toxin (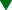) or PVL (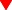). Leukotoxin HlgC/HlgB was the most active, showing a half-maximum concentration effect at nanomolar concentrations. The other three toxins triggered glutamate release at higher concentrations only; the glutamate measured in these instances was similar to the amount measured after a 10 s pulse of 60 mM KCl depolarization. B. Prior to addition of the toxin, cells were loaded with 10 μM of the membrane-permeable intracellular calcium chelator BAPTA-AM (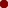), which led to a significant decrease in the amount of glutamate release. Control leukotoxin HlgC/HlgB (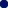). The upper reference value (6.51 μM, blue dotted line) stands for the total-cell glutamate content. C. Glucose-6-phosphate dehydrogenase enzymatic activity measured in culture media after a 10 min incubation of neurones in the presence of leukotoxin HlgC/HlgB. Only the highest concentration (0.1 μM) demonstrated a significant amount of released enzymatic activity. Results in (B) (glutamate) and (C) (G6PDH) are from of the same experiment. The brown dotted line represents glutamate (A and B) or G6PDH activity (C) measured in the bathing media of control cells. The green dotted line in (A) and (B) corresponds to glutamate released by a 10 s depolarization of neurones in 60 mM KCl. The blue dotted line in (B) and (C) stands for total glutamate (B) or total G6PDH (C) content measured after a 10 min osmotic shock of neurones in 10 mM Tri-HCl buffer (pH 7.4).

Cerebellar granular neurones have been shown to act in response to the presence of certain pore-forming toxins (Lonchamp *et al*., 2010). Thus, to examine whether neurones sense and react to *S. aureus* leukotoxins, glutamate release by cerebellar granular neurones was quantified after a 10 min exposure to the toxins. Dose–response curves for α-toxin, leukotoxins HlgC/HlgB and HlgA/HlgB and the PVL are shown in Fig. 1A. HlgC/HlgB was the most potent of the four leukotoxins, reaching a half-maximal effect at a concentration of 3 nM and maximum glutamate release at 0.1 μM. This maximum was 2.4 times higher than the total release generated by a 5 s depolarization pulse of 60 mM KCl, but only 35% of the total-cell glutamate content, as assessed using a 10 min osmotic shock in 10 mM Tris-HCl, pH: 7.4 (Fig. 1B). Alpha-toxin (0.1 μM), leukotoxin HlgA/HlgB (0.1 μM) and PVL (0.1 μM) were less effective than HlgC/HlgB, releasing equivalent amounts of glutamate as that generated by the 5 s 60 mM KCl depolarization pulse (Fig. 1A). The release due to leukotoxin HlgC/HlgB was dependent on an increase in free [Ca^2+^]_i_, since pre-incubation of neurones with the intracellular calcium chelator BAPTA-AM (10 μM) (30 min loading followed by 20 min hydrolysis) strongly reduced the amount of glutamate released into the medium (Fig. 1B). The HlgC or HlgB subunits separately failed to induce glutamate release from granular neurones (Fig. S1a).

To determine whether HlgC/HlgB-treated neurones undergo lysis, the amount of glucose-6-phosphate dehydrogenase (G6PDH) enzymatic activity released into the medium was quantified. An example of G6PDH activity measured after incubation at increasing concentrations of HlgC/HlgB leukotoxin is illustrated in Fig. 1C. Significant enzymatic activity was detected only after 20 min incubation with 0.1 μM leukotoxin, suggesting that high toxin concentration treatment may have been fatal for some cells. However, assessment of the number of pyknotic nuclei in cultures labelled with Hoechst-33258 revealed no significant difference between control (three independent cultures, > 400 nuclei, 6.5% pyknotic) and 6 nM leukotoxin-treated cells after a 15 min incubation period (three independent cultures, > 400 nuclei, 5.8% pyknotic). Membrane permeation of ethidium bromide has been proposed for monitoring pore formation at the cell membrane due to leukotoxin (Staali *et al*., 1998). Time-lapse video microscopy was used to record the infiltration of ethidium bromide in neurones incubated in the presence of 8 nM leukotoxin HlgC/HlgB-Alexa-488. Visual inspection of neurones incubated 30 min in the presence of toxin revealed that ethidium bromide-related fluorescence remained low, constant and consistently extracellular (Fig. S2). These results together with the reduction in glutamate release due to the Ca^2+^ chelator BAPTA favour the notion that leukotoxin-induced glutamate release is triggered by a rise in free [Ca^2+^]_i_, independently of the formation of a pore.

### The leukotoxin-induced rise in [Ca^2+^]_i_ is concentration dependent

**Fig. 2:**
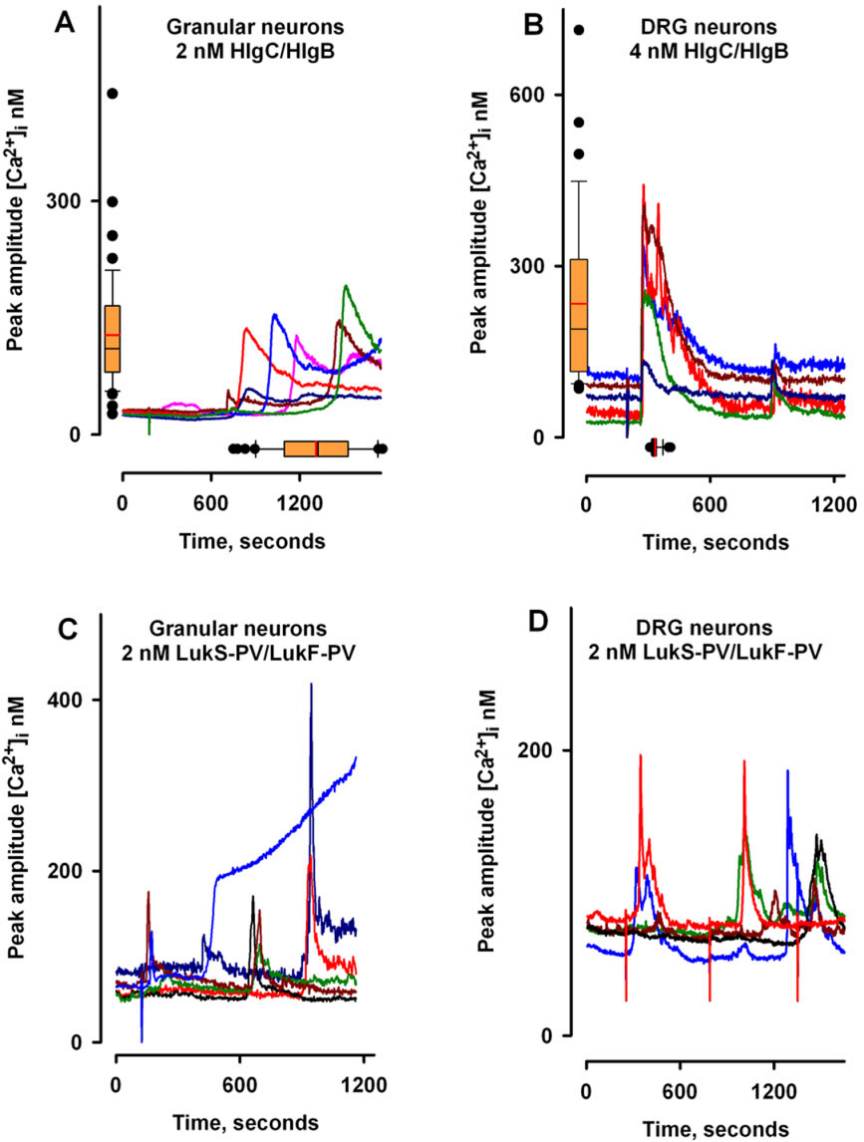
Leukotoxins induce [Ca^2+^]_i_ changes in cerebellar granular and in dorsal root ganglion neurones. A. Multiple examples of recordings of cerebellar neurones challenged with 2 nM leukotoxin HlgC/HlgB. The amplitude of the [Ca^2+^]_i_ peak induced by the leukotoxin was variable as was the latency of the response. Boxes along the axis show the median and percentiles for both peak amplitude and latency for 47 cells recorded simultaneously. The mean is indicated by the red trace. B. [Ca^2+^]_i_ variation caused by leukotoxin HlgC/HlgB in dorsal root ganglion neurones (4 nM toxin). The box presentation corresponds to 36 neurones from three different dishes recorded the same day. C. PVL (2 nM) induced [Ca^2+^]_i_ changes in 25% of recorded cerebellar neurones; in certain instances, the same cell presented a few [Ca^2+^]_i_ transient peaks. D. Responses of DRG neurones to different applications of 2 nM PVL; contrary to the γ-leukotoxin HlgC/HlgB effects, PVL was able to induce different responses within the same cell during the recording period. Less than 25% of DRG cells from the same culture plate were sensitive to the leukocidin. The vertical stroke at approximately 200 s indicates the time of addition of the toxin. In (D), the three vertical strokes indicate three independent additions of PVL. In (A)–(D), all traces represent individual sample recordings.

We monitored [Ca^2+^]_i_ variations in cerebellar granular neurones and in sensory neurones from dorsal root ganglia (DRG), using the Fura-2 Ca^2+^ indicator dye, to analyse the activity of leukotoxins HlgC/HlgB and HlgA/HlgB and PVL (Fig. 2). The subunits HlgC or HlgB separately failed to induce [Ca^2+^]_i_ variations (Fig. S1b). Leukotoxin HlgC/HlgB (2 nM) induced a rise in [Ca^2+^]_i_ in 86% of granular neurones (12 cultures, 618 cells, Fig. 2A) and in 65% of DRG neurones at 4 nM (9 cultures, 191 cells, Fig. 2B). In contrast, leukotoxin HlgA/HlgB activated a rise in [Ca^2+^]_i_ in 20% of granular neurones at higher concentration (20 nM; three experiments, 130 cells). PVL (2 nM) was active in 24% of recorded granular neurones (13 cultures, 419 cells, Fig. 2C) and in 28% of DRG sensory neurones at 4 nM (three cultures, 84 cells, Fig. 2D).

The typical reaction of a neurone to the presence of leukotoxin HlgC/HlgB was a progressive elevation in free [Ca^2+^]_i_ which led to a transient peak and returned to a new [Ca^2+^]_i_, resting value (Fig. 2A and B). In a same culture plate, the effect of the leukotoxin on different granular neurones occurred at various unrelated moments. However, the response of DRG sensory neurones was both fast and synchronous for all responding cells of the same plate. For comparison purposes, the response of each neurone was characterized by the peak value of [Ca^2+^]_i_ (amplitude) and the time needed by the cell to reach the [Ca^2+^]_i_ peak after the addition of the leukotoxin (latency). These values are shown in box plots with the mean, the median and percentiles.

**Fig. 3:**
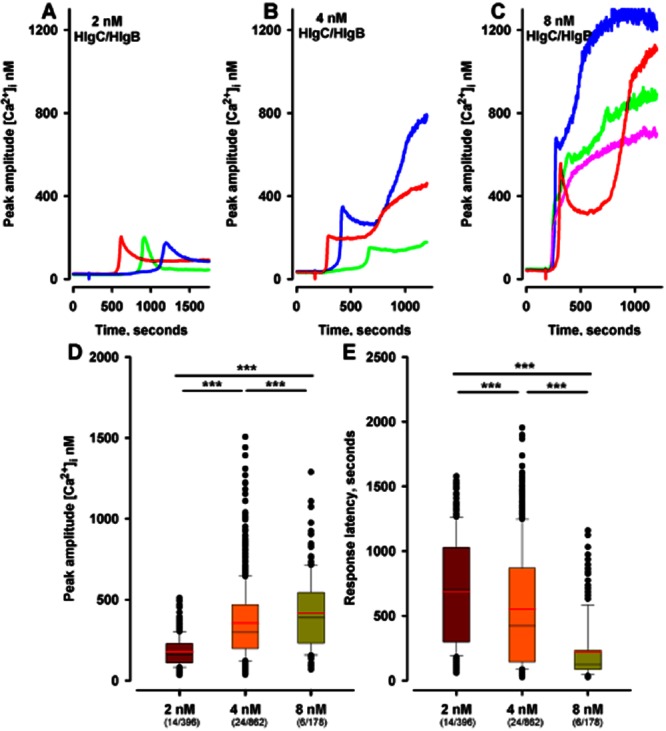
The mean of the [Ca^2+^]_i_ peak amplitude and the response latency to leukotoxin HlgC/HlgB are concentration dependent. A–C. Representative recordings of [Ca^2+^]_i_ changes in cerebellar neurones challenged with three different concentrations of leukotoxin HlgC/HlgB (A: 2 nM, B: 4 nM, C: 8 nM). The dot around 200 seconds in (A), (B) and (C) indicates the addition point of the toxin. These examples suggest a toxin concentration-dependent effect that was further established by pooling the values from 44 independent experiments. Boxes are the median and percentiles and include the mean value (red line). All other traces represent individual sample recordings. D and E. (D) [Ca^2+^]_i_ peak amplitude, (E) latency. The mean values are significantly different as determined by a Kruskal–Wallis analysis of variance and Dunn's Pairwise Multiple Comparison Procedures (***P < 0.005).

In granular neurones, the amplitude of the [Ca^2+^]_i_ peak was dependent on leukotoxin concentration, as illustrated in examples in Fig. 3A–C. Moreover, the consolidation of results from 44 independent experiments confirmed that mean [Ca^2+^]_i_ peak amplitude values increased, while the latency of the response decreased with increasing concentrations of leukotoxin, as shown in Table 1 and Fig. 3D and E. However, the dispersion of both parameters was high among neurones within a same plate, which suggests that a multistep process may be necessary to disrupt [Ca^2+^]_i_ homeostasis and that its activation may depend on a particular cell's status.

**Table 1:** Peak amplitude and latency values are dependent on toxin concentration

HlgC/HlgB	Mean [Ca^2+^] peak ± SEM	Latency, seconds ± SEM	Secondary [Ca^2+^] rise, % of cells
2 nM leukotoxin HlgC/HlgB (14 plates, 396 cells)	178 nM ± 4	687 s ± 21	
4 nM leukotoxin HlgC/HlgB (24 plates, 862 cells)	355 nM ± 8	551 s ± 15	54% cells
8 nM leukotoxin HlgC/HlgB (6 plates, 178 cells)	416 nM ± 17	221 s ± 18	98% cells

Mean values of the peak amplitudes and latencies of the experiment shown in Fig. 3D and E. Dunn's Pairwise Multiple Comparison shows that the differences among all mean values are statistically significant.

### The increase in free [Ca^2+^]_i_ is independent of voltage-operated Ca^2+^ channels or glutamate receptor activity

**Fig. 4:**
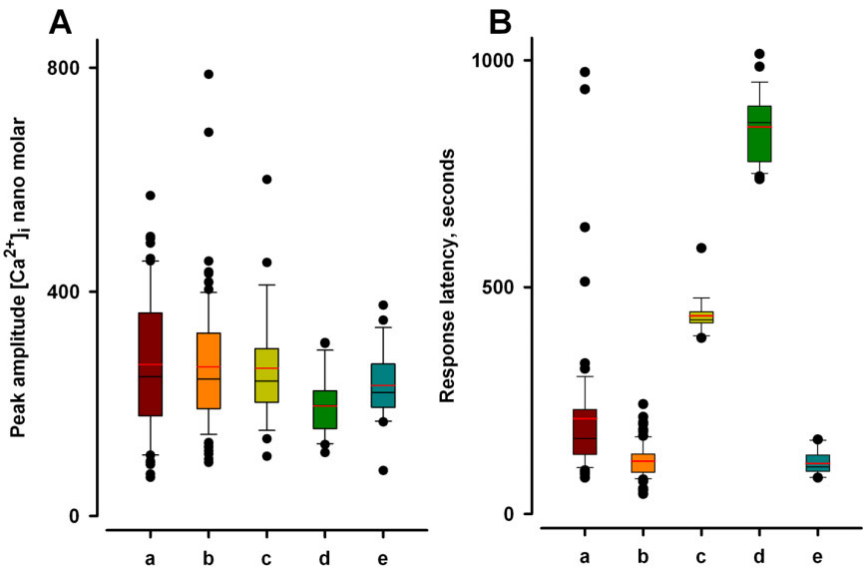
The rise in free [Ca^2+^]_i_ evoked by leukotoxins in cerebellar neurones is independent of voltage-operated Ca^2+^ channels or glutamate receptor opening. (A) Peak amplitude and (B) latency of recordings carried out in a: control (56 cells) or in the presence of HlgC/HlgB (4 nM) and the following blockers/antagonists: b: (5 μM nifedipine + 0.1 μM ω-GVI-A + 0.1 μM ω-Aga-TK; 88 cells), c: (0.3 μM TTX; 24 cells), d: (5 μM nifedipine + 0.1 μM ω-GVI-A + 0.3 μM TTX + 10 μM CNQX + 10 μM APV; 26 cells), e: (5 μM nifedipine + 0.1 μM ω-GVI-A + 0.1 μM ω-Aga-TK + 0.3 μM TTX + 10 μM CNQX + 10 μM APV; 21 cells). None of the differences in mean values for peak amplitude were statistically significant when examined by the Kruskal–Wallis analysis of variance. All the differences in mean latency values were statistically significant (*P* < 0.001) with respect to the control value (box a); pairwise multiple comparison showed a non-significant difference among values for b and e.

Activation of any of the various types of voltage-operated Ca^2+^ channels (VOCC) or glutamate receptors may lead to rise in [Ca^2+^]_i_ in granular neurones (Randall and Tsien, 1995; Brickley *et al*., 2001). To investigate whether leukotoxin HlgC/HlgB activates a Ca^2+^-permeable channel or receptor, VOCC were antagonized with either 5 μM nifedipine (L-Type channels), 0.1 μM ω-GVI-A (N-Type channels) or 0.1 μM ω-AgaTK (P/Q-Type channels) whereas ionotropic glutamate receptors were antagonized with 10 μM CNQX (AMPA receptors) or 10 μM D-AP5 (NMDA receptors) while recording [Ca^2+^]_i_ variations induced by leukotoxin HlgC/HlgB. Amplitude and latency of [Ca^2+^]_i_ variations are presented in Fig. 4A and B and in Table 2 (mean values). The presence of these drugs either alone or in various combinations, prior to addition of the leukotoxin, did not considerably alter the amplitude of the [Ca^2+^]_i_ peak (Fig. 4A). However, blockade of all VOCC significantly shortened the latency of the leukotoxin effect by 45% (*P* < 0.05), effect observed also when Na^+^ channels and glutamate receptors were additionally blocked (Fig. 4B, Table 2). When the P/Q-type Ca^2+^ channel antagonist ω-AgaTk was removed from the cocktail, the latency of the response was four times longer (853 ± 14 s versus 210 ± 20 s, *P* < 0.05). The selective blockade of voltage-gated Na^+^ channels (0.3 μM TTX) doubled the latency of the response (436 ± 8 s versus 210 ± 20 s, *P* < 0.05). Altogether, these results rule out both VOCC and glutamate receptors as direct passageways for extracellular Ca^2+^ influx induced by leukotoxin. However, given the impact of the blockade of these channels on the kinetics of the leukotoxin effect, a role for resting [Ca^2+^]_i_ or membrane potential cannot be excluded.

**Table 2:** Mean values and statistical significance of recordings with blunted voltage-operated Ca^2+^ channels (VOCC) and voltage-gated Na^+^ channels (VGNC)

8 nM HlgC/HlgB	Mean [Ca^2+^] peak ± SEM	Latency s ± SEM	Pairwise comparison of mean latencies (Holm-Sidak method)
a: control (65 cells)	270 ± 15 nM	210 ± 20 s	a versus b: *P* < 0.001; a versus c: *P* < 0.001; a versus d: *P* < 0.001; a versus e: *P* < 0.001
b: blockade of L-, N- and P/Q-Type VOCC by 5 μM nifedipine + 0.1 μM ω-GVI-A + 0.1 μM ω-Aga-TK (88 cells)	265 ± 12 nM	116 ± 4 s	b versus c: *P* < 0.001; b versus d: *P* < 0.001; b versus e: *P* = 0.839
c: blockade of VGNC by 0.3 μM TTX (24 cells)	263 ± 22 nM	436 ± 8 s	c versus d: *P* < 0.001; c versus e: *P* < 0.001
d: blockade of L- and N-Type VOCC, VGNC and Glu-R by 5 μM nifedipine + 0.1 μM ω-GVI-A + 0.3 μM TTX + 10 μM CNQX + 10 μM D-AP5 (26 cells)	196 ± 10 nM	853 ± 14 s	d versus e: *P* < 0.001
e: blockade of L-, N- and P/Q-Type, VGNC and Glu-R: 5 μM nifedipine + 0.1 μM ω-GVI-A + 0.1 μM ω-Aga-TK + 0.3 μM TTX + 10 μM CNQX + 10 μM D-AP5; 21 cells	232 ± 14 nM	111 ± 6 s	

Mean values and statistical significance of peak amplitudes and latencies of the experiment shown in Fig. 4. The pairwise comparison between the means of the peak amplitude did not reveal significant statistical differences, whereas mean latencies were significantly different, but for experiments b and e only.

**Fig. 5:**
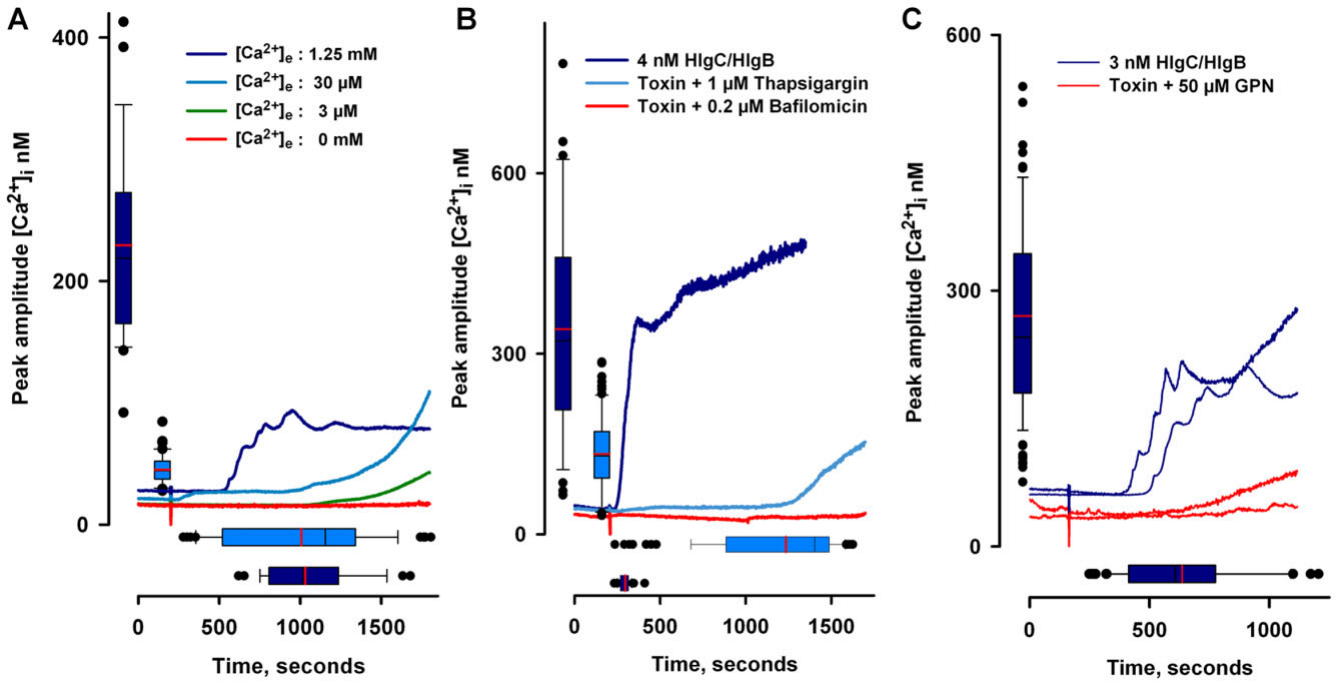
Both extracellular and intracellular Ca^2+^ stores contribute to leukotoxin-induced [Ca^2+^]_i_ changes in cerebellar neurones. A. Averaged traces of four recordings obtained at different extracellular Ca^2+^ concentrations ([Ca^2+^]_e_) showing that, in a Ca^2+^-free medium, neurones (35 cells) do not react to the presence of the leukotoxin. In low [Ca^2+^]_e_ (3 μM), an increase in free [Ca^2+^]_i_ can be observed (mean of 41 recorded neurones). Control recordings with 1.25 mM Ca^2+^ (34 cells) are shown as well as recordings with 30 μM Ca^2+^ (47 cells). The boxes show the distribution values for control and 30 μM Ca^2+^ recordings. B. Average traces of cells recorded upon interruption of reticular Ca^2+^ refilling by blockade of the SERCA pump (1 μM thapsigargin; two experiments, 66 cells) or upon interruption of acidic compartment Ca^2+^ refilling through the blockade of H-ATPase (0.2 μM bafilomycin; two experiments, 80 cells). The boxes show the distribution values for control and thapsigargin recordings. C. Lysosomal destruction by 0.2 μM Glycyl-1-phenylalanine 2-naphthylamide (GPN) prevented leukotoxin-induced [Ca^2+^]_i_ changes. Two different experiments are shown where the mean of control traces are compared with the mean traces of neurones recorded after addition of GPN. The boxes correspond to the values of control recordings (87 cells). In all panels, the addition point of the toxin is indicated by a vertical stroke.

To ascertain the contribution of extracellular Ca^2+^, neurones were equilibrated in buffers of various extracellular Ca^2+^ concentration ([Ca^2+^]_e_) prior to incubation with the leukotoxin HlgC/HlgB. As shown in Fig. 5A, the mean [Ca^2+^]_i_ peak was reduced by 80% in 30 μM [Ca^2+^]_e_ relative to controls (43 ± 2 nM versus 227 ± 7 nM; *n* = 50, *P* < 0.001), with no significant change observed for the latency of the effect. Leukotoxin-induced [Ca^2+^]_i_ changes could still be observed in neurones incubated in 3 μM [Ca^2+^]_e_ but not in cells bathing in Ca^2+^-free buffer (0 mM Ca^2+^, 5 mM EGTA), where resting [Ca^2+^]_i_ remained constant.

The contribution of Ca^2+^ stored in internal compartments was assessed by incubating the neurones in the presence of 1 μM thapsigargin (endoplasmic reticular stores) or 0.2 μM bafilomycin A (acidic stores) (Fig. 5B) prior to toxin application. In the presence of thapsigargin, 56% of the cells (mean of seven independent experiments) responded to the presence of leukotoxin HlgC/HlgB (4 nM) by displaying a transient peak of low amplitude (Fig. 5B: 160 ± 8 nM versus 362 ± 37 nM; *n* > 100, *P* < 0.001) whereas the latency of the response was prolonged (1447 ± 44 s versus 413 ± 6 s; *n* > 100, *P* < 0.001). Inhibition of vesicular H-ATPase by 0.2 μM bafilomycin A abolished nearly all leukotoxin-induced [Ca^2+^]_i_ movements; approximately half of the recorded cells showed delayed low-amplitude transient increases in [Ca^2+^]_i_ that barely reached twice the amplitude of resting [Ca^2+^]_i_. To confirm that Ca^2+^ release from acidic organelles indeed contributed to the leukotoxin effect, osmotic lysosomal degradation was induced with Glycyl-1-phenylalanine 2-naphthylamide (GPN) (Churchill *et al*., 2002). Application of GPN (200 mM, 10 min) essentially blunted the leukotoxin effect; only 11% of neurones produced low-amplitude transient variations in [Ca^2+^]_i_ or displayed a late and moderate increase in [Ca^2+^]_i_ after addition of the leukotoxin (three cultures, 87 cells, Fig. 5C). These observations suggest that Ca^2+^ released from acidic compartments may couple with reticular stores through a Ca^2+^-induced Ca^2+^ release (CICR) mechanism. Moreover, the contribution of Ca^2+^ from both the external medium and intracellular stores points towards the Store-Operated Ca^2+^ Entry (SOCE) complex as a final outcome of Ca^2+^ mobilization initiated by the leukotoxin.

### Leukotoxin HlgC/HlgB alters the store-operated Ca^2+^ entry complex

**Fig. 6:**
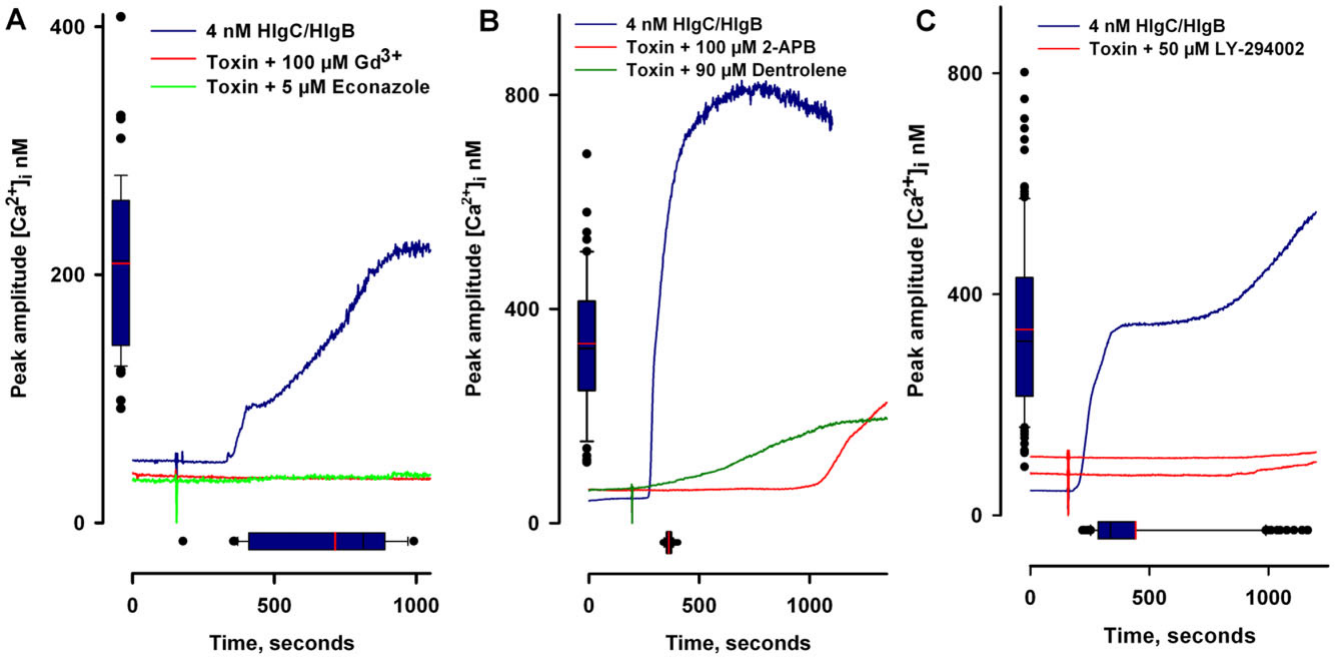
Disruption of the Store-Operated Ca^2+^ entry complex strongly inhibits the effect of leukotoxin HlgC/HlgB in cerebellar neurones. A. Mean traces of cells recorded in the presence of 5 μM econazole (24 cells) and 100 μM gadolinium (38 cells). The control experiment corresponds to the mean of 35 cells. B. Mean traces of cells recorded in the presence of drugs that target SOCE by also interfering with Ins(1,4,5)-P3 Ca^2+^ mobilization (2-APB, 100 μM) or ryanodine receptors (dentrolene, 90 μM). Pre-treatment with these agents significantly reduced the effect of γ-leukotoxin HlgC/HlgB. Control experiment, 38 cells; 2-APB, 13 cells; dentrolene, 49 cells. This experiment was reproduced three times. C. incubation of neurones in the presence of LY-294002 (50 μM), which inactivates phosphatidylinositol 3 and 4 kinase and modifies membrane PIP2 content, thus strongly affecting the cellular response to leukotoxin action. Control boxes present the median of 110 recorded cells and LY-294002 (red lines) recordings are the mean of 52 and 63 treated cells. The boxes in the three panels correspond to the values of control recordings. Addition of the toxins is indicated by a vertical stroke in all panels.

Neuronal [Ca^2+^]_i_ variations were monitored in the presence of drugs or ions known to interfere with SOCE (Bird *et al*., 2008; Varnai *et al*., 2009). A highly effective neutralization was observed when 100 μM Gd^3+^ was added a few minutes prior to the leukotoxin (Fig. 6A). At lower concentration, Gd^3+^ (50 μM) reduced the [Ca^2+^]_i_ amplitude by 44% (291 ± 20 nM versus 519 ± 20 nM; *n* > 50, *P* < 0.001) and slightly affected the time of the response, but totally abolished the second Ca^2+^ rise present in 96% of control cells challenged with 4 nM leukotoxin (not shown). The SOCE antagonist econazole (5 μM), totally prevented the toxin-induced rise in [Ca^2+^]_i_ (Fig. 6A). In addition, incubation of cells for 15 min in 100 μM 2-aminoethoxydiphenylborane (2-APB), a cell-permeable modulator of inositol (1,4,5)-P3-induced Ca^2+^ release targeting SOCE, decreased the peak amplitude by 40% (203 ± 18 nM versus 335 ± 17 nM; *n* > 20, *P* < 0.001) and increased the latency of the response by more than 10-fold (1149 ± 47 s versus 100 ± 1 s; *n* > 20, *P* < 0.001) (Fig. 6B). Cells maintained for 15 min in the presence of 90 μM dentrolene, which also blocks ryanodine and Ins(1,4,5)-P3 receptors, showed a 57% reduction in amplitude (144 ± 4 nM versus 335 ± 17 nM; *n* > 40, *P* < 0.001) and a sixfold delay in latency (608 ± 25 s versus 100 ± 1 s; *n* > 40, *P* < 0.001) (Fig. 6B). The pyrazole derivative YM-58483 has been reported as an inhibitor of the Ca^2+^ release-activated Ca^2+^ (CRAC) channels (Ishikawa *et al*., 2003). Pre-incubation of neurones in 10 μM YM-58483 significantly modified the leukotoxin effect by reducing the amplitude of the Ca^2+^ peak by 70% (142 ± 10 nM versus 458 ± 31 nM; *n* > 26, *P* < 0.001), while the latency of the effect remained unaltered. CRAC channels are formed in the plasma membrane by the association of Orai1 monomers, mediated by the reticular protein STIM1 that senses the ER Ca^2+^ filling state (Collins and Meyer, 2011 and references therein). The interaction between STIM1 and Orai1 is regulated by phosphatidylinositol 4,5-bisphosphate (PIP2) (Korzeniowski *et al*., 2009; Walsh *et al*., 2009). In order to verify whether the disruption of the STIM1–Orai1 interaction alters the leukotoxin effect, experiments were conducted in which phosphatidylinositol kinases (PI3K and PI4K) were inhibited by LY-294002 (50 μM, 15 min), thereby potentially modifying the PIP2 content, while recording leukotoxin-induced [Ca^2+^]_i_ changes. Under these conditions, only 6% of the 176 recorded cells (three independent experiments) responded to the leukotoxin through a low amplitude rise in [Ca^2+^]_I_ whereas in controls, 90% of the 155 recorded cells mobilized [Ca^2+^]_i_ (Fig. 6C).

**Fig. 7:**
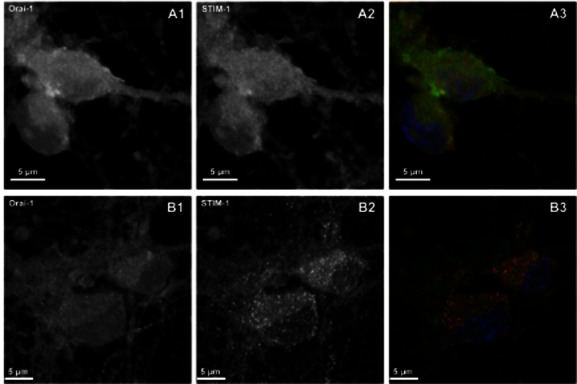
Distribution of STIM1 and Orai1 molecules of the SOCE complex before and after incubation of neuronal cells with 4 nM HlgC/HlgB-Alexa 488. Confocal acquisition images of cerebellar neurones labelled with the anti-Orai1goat polyclonal antibody D15 (A1, B1) and the anti-Stim1 mouse monoclonal antibody A-8 (A2, B2). Panels A3 and B3 correspond to merged images with Hoechst 33258-labelled nuclei. Cells in row A were fixed prior to incubation for 10 min in the presence of 4 nM leukotoxin HlgC/HlgB; cells in row B were fixed after incubation with the toxin.

Following the Ca^2+^ depletion, the translocation of STIM1 molecules to endoplasmic reticulum–plasma membrane junctions activates the Orai1-formed CRAC channels; simultaneously the formation of STIM1 puncta can be observed using fluorescence labelling methods (Walsh *et al*., 2009). The consequences of leukotoxin HlgC/HlgB activity on the redistribution of interacting partners of the SOCE complex were investigated through immunolabelling of neurones with antibodies raised against STIM1 and Orai1. Figure 7 illustrates the differences in labelling observed for STIM1 in control cells relative to cells incubated for 10 min with 4 nM leukotoxin. In treated cells, labelling became partially punctuated suggesting that a redistribution of STIM1 is also an effect of leukotoxin HlgC/HlgB. Taken together, the above results from pharmacological manipulation as well as immunolabelling allow to decompose [Ca^2+^]_i_ movements induced by the leukotoxin as a succession of events beginning by the release of acidic Ca^2+^ stores and resulting in the stimulation of the SOCE complex.

### The HlgC/HlgB-induced rise in [Ca^2+^]_i_ is initially dependent on ADP-ribosyl cyclase CD38 signalling

**Fig. 8:**
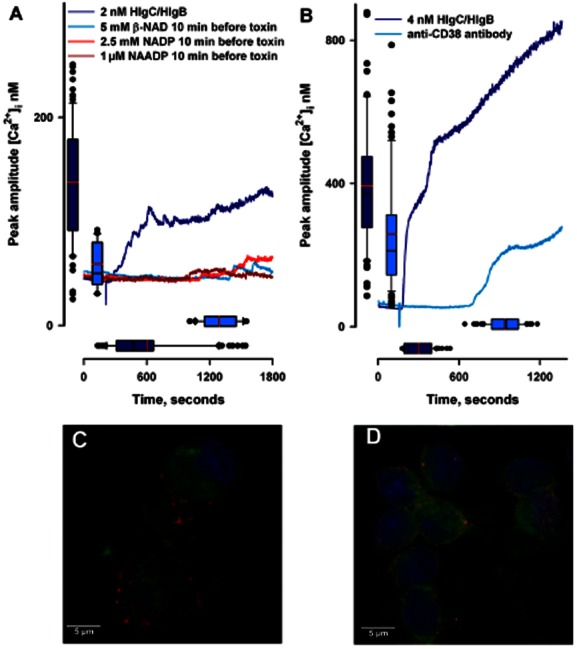
Leukotoxin-induced [Ca^2+^]_i_ movements in cerebellar neurones require ADP-ribosyl cyclase CD38 activity. A. CD38 internalization by addition of 5 mM β-NAD prior to adding leukotoxin HlgC/HlgB significantly reduced its effect. The results combine data from three independent experiments. Control line and boxes (101 cells); recordings of β-NAD pre-treated cells are the mean of the 25% responding cells. Recordings of cells pre-treated with 2.5 mM or 1 μM NAADP are also shown. The boxes represent control and 5 mM β-NAD recordings. B. Effect of incubation of granular neurones in the presence of the anti-CD38 goat polyclonal antibody prior to addition of 4 nM leukotoxin. The decrease in mean peak amplitude and the reduction in response latency were statistically significant (*P* < 0.001). Control, 190 cells; CD38 antibody, 94 cells. C. Granular neurones labelled with HlgC/HlgB-Alexa 488 and Cholera toxin-B-subunit-Alexa 594 conjugated. D. Neurones labelled with HlgC/HlgB-Alexa 488 and anti-CD38 goat polyclonal antibody M-19 revealed by a donkey anti-goat Alexa 594-conjugated secondary antibody.

We next focused on the early signalling which could be activated by the leukotoxin in the plasma membrane and induce the release of Ca^2+^ from acidic stores. Lysosomes release Ca^2+^ through two-pore channels that are opened by nicotinic acid adenine dinucleotide phosphate (NAADP), an intracellular messenger produced by the ADP-ribosyl cyclase CD38 (Zhu *et al*., 2009; Cosker *et al*., 2010). Although efficient antagonists of CD38 activity are not currently available, it is known that β-NAD rapidly induces the internalization of the enzyme and reduces its activity (Zocchi *et al*., 1999). Hence, we assessed the potential effect of preventing granular neurones from being activated by the leukotoxin by incubating the latter for 10 min in the presence of 5 mM β-NAD prior to the addition of the toxin. In four independent experiments, only 26% of recorded cells showed a transient rise in [Ca^2+^]_i_, along with a significant reduction in mean peak amplitude (191 ± 14 nM versus 444 ± 18 nM; *n* = 23, *P* < 0.001) and a delayed latency (1147 ± 31 s versus 537 ± 35 s; *n* = 23, *P* < 0.001) (Fig. 8A). Similar effects were observed using 2.5 mM NADP or 1 μM NAADP added 10 min prior to the toxin (Fig. 8A). NAADP likely induces a desensitization of the lysosomal two-pore channel (Zhu *et al*., 2009) that may prevent leukotoxin signalling. Challenge of the granular neurones by 5 mM β-NAD or 2.5 mM NADP did not produce [Ca^2+^]_i_ peaks, but induced a slight increase in [Ca^2+^]_i_ level.

The enzymatic properties of CD38 can be altered by antibodies directed against its C-terminal portion (Ferrero *et al*., 2004; Hara-Yokoyama *et al*., 2008). When cells where incubated for 20 min in the presence of an anti-CD38 C-terminal goat polyclonal antibody (M-19 antibody, Santa Cruz Biotechnology), the response to the leukotoxin was significantly delayed (842 ± 25 s versus 378 ± 27 s; *n* = 94, *P* < 0.001) along with a reduction in the peak of [Ca^2+^]_i_ (259 ± 16 nM versus 430 ± 18 nM; *n* = 94, *P* < 0.001) (Fig. 8B).

Granular neurones incubated in Alexa 488-tagged HlgC/HlgB leukotoxin (4 nM) were observed by confocal microscopy after fixation and paired labelling the anti-CD38 antibody or Alexa 594-conjugated Cholera toxin-B subunit (Fig. 8C and D). The anti-CD38 labelling was observed at the edge of the cells, showing a dotted aspect that partially appeared superimposed with leukotoxin labelling. However, the fluorescence of Alexa 488-HlgC/HlgB leukotoxin appears located preferentially in the cytosol. Labelling with the Alexa 594-Cholera toxin, which binds membrane gangliosides, surrounded the cells and was clearly segregated from the Alexa 488-leukotoxin fluorescence. These results suggest that the leukotoxin may be partially trapped in intracellular organelles together with CD38.

**Fig. 9:**
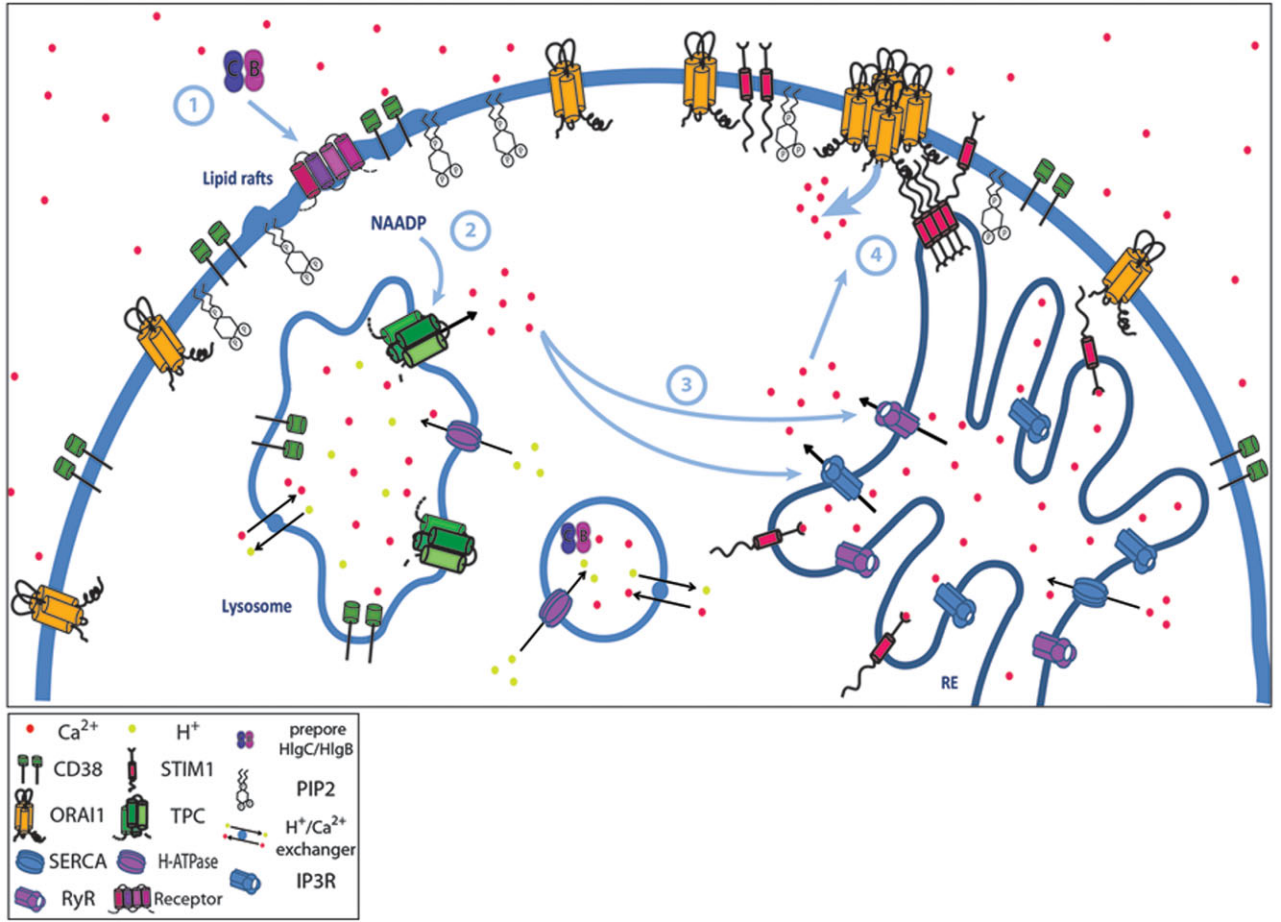
Schematic representation of the multistep signalling mechanism triggered by γ-leukotoxin HlgC/HlgB on neuronal cells. Upon binding (1) to a receptor complex, likely associated with lipid rafts, the bi-component leukotoxin triggers the formation of NAADP by ADP-ribosyl cyclase CD38. NAADP, acting on the two-pore Ca^2+^ channels of lysosomes (2) elicits the release of Ca2+ into the cytosol. Free [Ca^2+^]_i_ can further be increased (3) through a Ca^2+^-induced Ca^2+^ release mechanism from reticular stores. Finally, reticular Ca^2+^ depletion activates the Store-Operated Ca^2+^ Entry complex formed by STIM1 and Orai1 (4).

Taken together, the results presented here can be better understood if confronted to the mechanism proposed for the endolysosomal Ca^2+^ signalling in a variety of cells (Morgan *et al*., 2011). It is suggested that NAADP initiates a multistep process by releasing Ca^2+^ from acidic stores, which triggers the further release of reticular Ca^2+^ and finally causes the activation of CRAC channels. The HlgC/HlgB leukotoxin could initiate the same signalling pathway, as we suggest through the schematic representation of Fig. 9.

## Discussion

### *S. aureus* leukotoxins are a critical threat to the nervous system

*Staphylococcus aureus* has been shown to efficiently invade human brain microvascular endothelial cells, which results in tissue damage, brain abscesses and meningeal inflammation (Pedersen *et al*., 2006; Sheen *et al*., 2010). We carried out this work in cerebellar granular neurones to assess whether leukotoxins may cause damage to nervous tissue. Rat granular neurones have been used for cellular studies on bacterial toxins while glutamate release assays have aided in investigating their neurotoxicity (Foran *et al*., 2003; Lonchamp *et al*., 2010). The use of these cells allowed us to establish that different *S. aureus* leukotoxins can represent a serious threat to neurones by inducing a rise in free intracellular Ca^2+^ and, as a consequence, the release of glutamate. The increase in free [Ca^2+^]_i_ was also measured in DRG neurones, highlighting the fact that the activity of leukotoxins is not restricted to a single class of neurones. The γ-haemolysin HlgC–HlgB was found to be the most potent, both in cerebellar and in DRG neurones, inducing important cellular effects while preserving the integrity of the plasma membrane. The pharmacological manipulations, which can prevent free [Ca^2+^]_i_ movements through the disruption of internal stores as well as acting on the plasma membrane, together with the lack of permeability to ethidium bromide, parallel to the [Ca^2+^]_i_ increase, strongly supports the notion that the formation of a pore is not the first cellular effect induced by leukotoxins in neurones. Cellular differences between neurones and neutrophils may explain an expanded delay between intracellular Ca^2+^ rise and pore formation as it has been observed in neutrophils through the introduction of mutations in PVL (Baba Moussa *et al*., 1999). Moreover, PVL can confer an enhanced bactericidal capacity to human neutrophils at very low concentrations, independently of the formation of a pore (Graves *et al*., 2012). The activity of the HlgC/HlgB leukotoxin is concentration dependent, which may suggest that minute amounts of toxin can be sensed by neurones and induce intracellular signalling. A neuronal early response to leukotoxin could have the positive outcome of initiating cellular protection (Aroian and van der Goot, 2007), conceivably in association with noxious stimuli.

Neurones preferentially use VOCC and ionotropic neurotransmitter receptors as Ca^2+^ influx pathways, while for neutrophils the SOCE complex is the main Ca^2+^ signalling system. This difference could obscure the fact that both cells types are sensitive to the leukotoxin. Interestingly, the Ca^2+^ activated movement shown herein demonstrates a reasonable link between internal Ca^2+^ stores and membrane signalling proteins, through pathways which are otherwise observed separately in neurones. The ADP-ribosyl cyclase CD38 has been detected in rat brain (Yamada *et al*., 1997; Ceni *et al*., 2003), where the signalling molecule NAADP has been proposed as a stimulator of neurite outgrowth (Brailoiu *et al*., 2005). The release of Ca^2+^ from either lysosomes or Ryanodine-sensitive pre-synaptic stores has been shown to enhance the efficiency of neurotransmitter release (Collin *et al*., 2005; McGuinness *et al*., 2007). In cortical and hypocampal neurones, differential regulatory roles of intracellular Ca^2+^ levels have been observed for STIM1 and STIM2 (Gruszczynska-Biegala *et al*., 2011), and the depletion of Orai1 impairs the rhythmic firing flight motoneurones in drosophila (Venkiteswaran and Hasan, 2009). A further question will be to ascertain whether endogenous activator(s) would be able to stimulate this ‘leukotoxin signalling multistep process’ in the nervous system. The future identification of the molecular complex that binds the leukotoxins will help to find the answer, as it will contribute to assess the consequences of the Ca^2+^ imbalance caused by leukotoxins in neurones. It is of interest to note that in mild *S. aureus* pathologies, such as chronic rhinosinusitis, associated headaches and facial pain/pressure negatively affect quality of life and impair daily activities (Singhal *et al*., 2011).

### A multistep Ca^2+^ signalling mechanism that could be shared by other toxins

An increase in free intracellular Ca^2+^ has already been described as an early response for the activity of different pore forming toxins. Intracellular Ca^2+^ movements are regarded as a prerequisite for the pre-pore to pore transition of membrane bound bi-component *S. aureus* leukotoxins (Woodin and Wieneke, 1963; Staali *et al*., 1998). The α-haemolysin from *Escherichia coli* has been shown to induce [Ca^2+^]_i_ oscillations in renal epithelial cells that are sensitive to L-type Ca^2+^ channel blockers (Uhlen *et al*., 2000), although the contribution of the L-type channel was subsequently challenged (Koschinski *et al*., 2006). In the present study, the pharmacological neutralization of voltage-operated Ca^2+^ channels, including L-type channels, as well as glutamate receptors revealed that these channels are not a primary pathway for the *S. aureus* leukotoxin-induced rise in [Ca^2+^]_i_. However, the latency to reach the [Ca^2+^]_i_ peak was affected by combinations of drugs known to be active on voltage-operated channels, which may suggest an influence of either the resting [Ca^2+^]_i_, or the membrane potential, on the kinetics of leukotoxin activity. The vacuolating cytotoxin A (VacA) from *Helicobacter pylori* induces the oscillation of free cytosolic Ca^2+^ through the mobilization of internal stores (De Bernard *et al*., 2005). The leukotoxin HlgC–HlgB shares the feature of mobilizing internal stores. Its activity was significantly inhibited when reticular Ca^2+^ stores were depleted. Even though the total reticular Ca^2+^ load remains low, and free cytosolic [Ca^2+^]_i_ slightly changes upon blocking the SERCA pump with thapsigargin (Pinilla *et al*., 2005), the reticulum is an important link for the response process activated by the toxin. A drastic inhibition was observed when H-ATPase, which contributes to the refilling of acidic Ca^2+^ stores, was blocked by Bafilomycin A, or upon disruption of lysosomes by GPN. Therefore, we propose that an initial release of Ca^2+^ from the acidic stores may be followed by a Ca^2+^-induced Ca^2+^ release from the endoplasmic reticulum. The Ca^2+^ depletion of the endoplasmic reticulum, sensed by the stromal interacting molecule STIM1, subsequently leads to the assembly of Orai1 molecules to form the store-operated CRAC channel (Varnai *et al*., 2009). Noteworthy, listeriolysin O, produced by *Listeria monocytogenes*, mobilizes Ca^2+^ from the extracellular milieu as well as from various types of intracellular stores (Gekara *et al*., 2007) whereas Streptolysin O, from *Streptococcus pyogenes*, induces long-lasting intracellular Ca^2+^ oscillations through the activation of the SOCE complex, which are modulated by the level of expression of STIM1 and Orai1 (Usmani *et al*., 2012). Another leukotoxin, secreted by *Actinobacillus actinomycetemcomitans,* induces free [Ca^2+^]_i_ movements if the molecules needed for its activity are previously clustered into lipid rafts (Fong *et al*., 2006). In the present study, the inhibition of *S. aureus* leukotoxin activity through LY294002 inactivation of PI3K and PI4K may suggest a similar prerequisite for leukotoxin HlgB/HlgC. However, the relationship between membrane PiP2 content and the regulation of SOCE (Korzeniowski *et al*., 2009; Walsh *et al*., 2009), could also be an alternative explanation for such inhibition.

The contribution of intracellular acidic stores as an early step of Ca^2+^ mobilization induced by a bacterial toxin is a novel finding. It is worth mentioning that signalling molecules linking plasma membrane receptors to the release of Ca^2+^ from internal stores are now starting to be identified (Cosker *et al*., 2010). The more potent of these molecules is NAADP, which is synthesized by the ADP-ribosyl cyclase CD38 (Malavasi *et al*., 2008) and activates the two-pore Ca^2+^ channels from lysosomes (Zhu *et al*., 2009). Our various approaches in shutting down CD38 activity by mimicking published protocols (Zocchi *et al*., 1999; Ferrero *et al*., 2004; Hara-Yokoyama *et al*., 2008), successfully reduced or suppressed the leukotoxin effect, strengthening the probable relationship between leukotoxin HlgC/HlgB binding and Ca^2+^ release from acidic stores. Summarizing the pharmacological manipulations that alter the granular neurones response to HlgC/HlgB leukotoxin, Fig. 9A illustrates a multistep process evoked by the endolysosomal Ca^2+^ signalling (Morgan *et al*., 2011). The cell membrane molecule (or molecules) that bind the toxin needs to be identified; here is represented by a ‘schematic receptor’. The step ‘binding’ (1) must be connected to the activation of the CD38 ADP-ribosyl cyclase, since the toxin activity is significantly inhibited by β-NAD acting on CD38 or the action of an anti-CD38-specific antibody. Step 2 comprises the NAADP mediated activation of Ca^2+^ release from the endolysosomal compartment, as suggested by the total inhibition caused by Bafilomycin A or GPN. The local release of Ca^2+^ triggers further Ca^2+^ release from the thapsigargin-sensitive reticular store (step 3). The final step (4) involves the activation of CRAC channels induced by the Ca^2+^ reticular stores depletion.

It would be of interest to determine whether other bacterial toxins mobilizing intracellular Ca^2+^ initiate a related mechanism, regardless of other effects they may induce. Similarly to *S. aureus* bi-component leukotoxins that evoke intracellular Ca^2+^ rise in neurones, even though they are known to discriminate between target cells (Menestrina *et al*., 2003). Irrespective of their similarities, the γ-haemolysins HlgA–HlgB and HlgC–HlgB share their F-component, the S-components HlgC and LukS-PV can compete for a same binding site (Prévost *et al*., 1995; Meyer *et al*., 2009), *S. aureus* leukotoxins share an initial intracellular Ca^2+^ mobilization which may induce a variable final outcome depending on the cell type or on the specific toxin. A question to focus on will be how bi-component bacterial toxins initiate cell signalling. It is tempting to speculate about some multi-molecular system, which should integrate the characterized cellular responses that are independent of the pore forming ability, such as cytotoxicity (Genestier *et al*., 2005; Zivkovic *et al*., 2011), enhanced bactericidal capacity (Graves *et al*., 2012) and Ca^2+^ mobilization from acidic stores. A comparative study of the responses of cultured neurones, alveolar macrophage and neutrophils to the *S. aureus* bi-component leukotoxins would be of valuable interest.

## Experimental procedures

### Ethic statement

All experiments have been conducted using protocols designed according to the European and French guidelines on animal experimentation and approved by the direction of the Bas-Rhin veterinary office, Strasbourg, France; authorization number 67-312 to E.J.

### Animals

Sprague-Dawley rats were obtained from the Institut des Neurosciences Cellulaires et Intégratives (INCI) animal facilities (French government agreement No. B-67-482-25). All procedures regarding housing, feeding and sacrifice of the animals were in accordance with approved European guidelines (No. 86/609/CEE) on animal care and experimentation.

### Toxins

The *S. aureus* α-toxin, γ-haemolysins HlgC/HlgB and HlgA/HlgB and the Panton – Valentine leukocidin LukS-PV/LukF-PV where purified as described previously (Werner *et al*., 2002). The fluorescent derivatives for HlgB were prepared as previously described (Finck-Barbançon *et al*., 1991).

### Drugs and chemicals

All standard chemicals, the glutamate receptor antagonists CNQX and APV, the calcium blockers and modulators nifedipine, dantrolene, 2-Aminoethoxydiphenyl borate D-AP5, Bafilomycin A1, Glycyl-1-phenylalanine 2-naphthylamide and the PI3Kinase antagonist LY-294002 were purchased from Sigma-Aldrich (Saint-Quentin Fallavier, France). *N*-[4-[3,5-*Bis*(trifluoromethyl)-1*H*-pyrazol-1-yl]phenyl]-4-methyl-1,2,3-thiadiazole-5-carboxamide (YM-59483) was from Tocris Bioscience (Bristol, UK). The voltage-gated channel blockers Tetrodotoxin, ω-Conotoxin GVI-A, ω-Agatoxin TK as well as ryanodine and thapsigargin were from Alomone Labs (Israel). Fura2-AM was from TEFLabs (Austin, TX), while Pluronic-127 and BAPTA-AM were from Molecular Probes/Life Technologies (Saint Aubin, France). Stock solutions of the drugs were prepared in their appropriate solvent (ethanol, dimethyl sulfoxide or water) at 1000× the final working concentration. The Ca^2+^-free bath solution used in selected experiments was prepared by omitting CaCl_2_ from the standard solution and adding 10 mM EGTA.

### Cerebellar granular neurones

Cell culture media and reagents, enzymatic solutions and antibiotics were from Gibco/Life Technologies (Saint Aubin, France). Insulin, progesterone, putrescine and human apo-transferrin were from Sigma (Saint Quentin Fallavier, France). Cerebellar cortices from postnatal day 5 (P5) rats were incubated for 5 min at 37°C in trypsin 0.05%–EDTA 0.02% solution, followed by 20 min at 37°C in papain 10 U ml^−1^ and DNase (250 U ml^−1^). Enzymes were inactivated by adding fetal calf serum (10%) and removed by three successive washes in culture media. The tissue was mechanically dissociated through polished Pasteur pipettes of decreasing tip diameter. After removal of tissue debris, cells were centrifuged and the pellet resuspended in culture medium. Cells were seeded on either poly-l-lysine-coated (10 μg ml^−1^) 24-well plates (Corning) at a density of 3.5 × 10^5^ cells per well for glutamate release studies, in 35 mm glass bottom Petri dishes for microfluorimetry, or on 11-mm-diameter glass coverslips for immunocytochemical studies. Cells were then incubated at 37°C in a 5% CO_2_-air humidified atmosphere. The culture medium consisted of B27-supplemented Neurobasal medium (Gibco/Life Technologies) and 25 mM KCl, 2 mM l-glutamine and penicillin/streptavidin antibiotics. Growth medium was further complemented with 1 μM insulin, 20 nM progesterone, 0.1 mM putrescine, 80 μg ml^−1^ transferrin and 0.5 μM sodium selenite. Cultures were also supplemented with 5 μM cytosine β-d-arabinofuranoside 24 h after plating in order to prevent glial proliferation; culture media were renewed every 3 days. Experiments were performed after 8-day differentiation *in vitro* to allow neuronal stabilization of intracellular Ca^2+^ stores (Mhyre *et al*., 2000).

### Dorsal root ganglion neurones

Dorsal root ganglia from as many spinal levels as possible were collected from euthanized P5 rats. The cells were dissociated from the ganglia as described previously (Jover *et al*., 2005). The cells were seeded on poly-l-lysine-coated bottom-glass Petri dishes at a density of 1 × 10^5^ cells per dish. Culture media consisted of complete Neurobasal medium supplemented with 100 ng ml^−1^ Nerve Growth Factor (Alomone Labs, Israel). The culture medium was renewed 4 days after plating. Experiments were performed using neurones cultured for 4–7 days.

### Glutamate release and quantification

Prior to toxin incubation or KCl stimulation, the medium was removed and the cell monolayer washed three times in Hanks balanced salt solution (HBSS) pre-warmed at 37°C. The appropriate toxin concentration was added for an incubation of 10 min. For KCl stimulation, cells were maintained in the depolarizing buffer (same as incubation buffer but with 60 mM KCl and 73 mM NaCl) for less than 10 s, before collecting the media for glutamate determination.

Glutamate cycling assay was based on the Amplex Red Glutamic Acid method (Chapman and Zhou, 1999). Typically, reactions were performed in 96-well plates (Falcon). To 50 μl of samples was added an equal volume of reagent mix containing 0.25 U ml^−1^ HRP, 0.08 U ml^−1^ glutamate oxydase, 0.5 U ml^−1^ glutamate pyruvate synthetase, 0.2 mM l-alanine and 100 μM 10-acetyl-3,7-dihydroxyphenoxazine (AnaSpec, Fremont, CA) in 0.1 mM Tris-HCl buffer pH 7.4. The cycling reaction was allowed to proceed at 37°C for 30 min and the increase in resorufin fluorescence was measured using a Mithras LB 940 fluorescence plate reader (Berthold Technologies, Thoiry, France) at 530 nm excitation and 590 nm emission.

Results are expressed as means ± SEM from at least four independent well measurements for each concentration of toxin. Glutamate determination was performed in triplicate. Statistical significance was determined by one-way analysis of variance followed by a Student's *t*-test using SigmaPlot for Windows Version 11.0. Differences were considered significant at *P* < 0.05.

### Glucose-6-phosphate dehydrogenase

Quantification of cell death was performed using the fluorescence-based micro-plate assay (Batchelor and Zhou, 2004) which measures glucose-6-phosphate dehydrogenase (G6PD) activity released into the media. The same supernatant collected for glutamate determination was also used for G6PDH activity. Reactions were conducted in a 100 μl final volume (96-well plates). Experimental samples were mixed 1:1 (v/v) with reagent mix containing 15 μM resazurin, 2 mM Glucose-6-Phosphate, 0.5 mM NADP and 0.5 U ml^−1^ diaphorase in 100 mM Tris-HCl buffer, pH 7.5. The reaction was carried out at 37°C for 30 min and the increase in fluorescence was measured as described for the glutamate assay.

### Immunocytochemistry

The subcellular localization of leukotoxin in granular neurones was detecting using the HlgB subunit tagged with Alexa Fluor 488 C_5_ maleimide (Life Technologies). Cells were incubated at room temperature for 1–10 min in the presence of 1 nM native or 5 nM tagged HlgC/HlgB. Neurones were then fixed in 4% (v/v) paraformaldehyde and 4% (w/v) sucrose in phosphate-buffered saline (PBS) for 15 min at room temperature and subsequently rinsed with PBS. Neurones were permeabilized and non-specific epitopes were blocked in PBS buffer containing 0.1% (v/v) Triton X-100, 1% (w/v) bovine serum albumin and 5% (v/v) normal goat serum (Chemicon-Millipore, Molsheim France) for 30 min prior to 2 h incubation at room temperature with antibodies diluted in the same buffer. The following primary antibodies were used: the anti-STIM1 (N-19) goat polyclonal antibody and A8 mouse monoclonal antibody; the anti-mouse CD38 (2Q1628) rat monoclonal antibody and M-19 goat polyclonal antibody (Santa Cruz Biotechnology). The plasma membrane was labelled by the cholera toxin B subunit conjugated to Alexa Fluor 594 (Life Technologies). After three washes in PBS, secondary antibodies tagged with Alexa 488 or Alexa 546 (Molecular Probes/Life Technologies, 1:1000) were incubated in PBS for 2 h at room temperature. After the immunostaining process, cell nuclei were stained using Hoechst 32258 (Sigma) at a final concentration of 40 nM. After washing in PBS, coverslips were stored at 4°C until observation. A Leica SP5-II confocal microscope was used.

### Microfluorimetry

Intracellular [Ca^2+^] recordings were performed on cells grown on glass coverslips and loaded with 5 μM Fura-2 AM – 0.04% Pluronic 127 for 30 min in the dark at 37°C, washed twice in HBSS and transferred to an inverted epifluorescence microscope (Axiovert, Zeiss, Germany) equipped with a UPlanFL 40/0.75 objective. The cells were alternatively illuminated at 350 nm and 380 nm and image pairs of the 520 nm light emission were recorded every 2 s for 30 min. Cells were bathed in HBSS buffer containing 10 mM Hepes buffer (pH 7.2) or, if needed, continuously superfused with the same buffer at a rate of 1 ml min^−1^. The toxins were applied directly into the bath. For DRG neurones, a solution of ATP (1 μM final concentration) preceded of 1 min the administration of the toxin; this ATP application never induced [Ca^2+^]_i_ changes. The ratio of fluorescence intensities (Ex350 nm/Ex380 nm) was calculated on a pixel basis for each image pair. The calcium concentration was then estimated by the formula (Grynkiewicz *et al*., 1985):


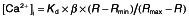


where *K*_d_ is the dissociation constant of Fura-2 for Ca^2+^ (924 nM, calculated in our cultures), *β* = (*I*_380 max_)/(*I*_380__min_) (2.08 ± 0.03, *n* = 39). Values of *R*_min_ and *R*_max_ were determined by periodical calibrations (Grynkiewicz *et al*., 1985).

### Statistics

Data are presented as box-plots with median, 10th, 25th, 75th and 90th percentiles and the outlying points; the mean line is also shown. Statistical significance was determined by Kruskal–Wallis one-way analysis of variance (SigmaPlot for Windows version 11.0). Differences were considered significant at *P* < 0.05.

## Supporting information

**Fig. S1.** The HlgC or HlgB subunits separately fail to induce glutamate release or free intracellular [Ca^2+^] changes in granular neurones.

a. Dose–response curves of glutamate released by granular neurones challenged for 10 min with the indicated concentrations of leukotoxin HlgC/HlgB (), HlgC subunit alone () or HlgB subunit alone (). The green line shows the glutamate measured after a 10 s pulse of 60 mM KCl depolarization.

b. averaged traces of records obtained in the presence of 2 nM HlgC/HlgB leukotoxin (51 cells, blue line), 2 nM HlgC subunit alone (60 cells, green line) and 2 nM HlgB subunit alone (31 cells, dark red line). The boxes show the distribution values of [Ca^2+^] peak amplitude (vertical box) and peak latency (horizontal box) from the control records.

**Fig. S2.** Confocal microscopy records of living granular neurones incubated in 12 μM Ethidium bromide and Leukotoxin HlgC/HlgB. Optical sections of 1.68 μm of cells bathing in HBSS containing Ethidium bromide (12 μM) were observed on a Leica SP5-II confocal microscope (fields of magnification × 63, initial pixel size 79.4 nm). Images of 1024 × 1024 pixels were acquired using 500–545 nm (green channel), 589–750 nm (red channel) and Nomarsky-DIC. Line 1: sample images after 3 min incubation in 12 μM Ethidium Bromide; 1A shows the living cells autofluorescent signal in the green channel, 1B is the Ethidium Bromide red fluorescence signal and 1C shows the recorded cells. Line 2 shows the same cells 15 min after the addition in the bath of HlgC/HlgB-Alexa-488 tagged leukotoxin (4 nM final); 2A green channel, 2B red channel and 2C Nomarsky-DIC image. Line 3 shows the same two cells after 20 more min incubation in 8 nM final concentration of HlgC/HlgB-Alexa-488 tagged; 3A green fluorescence, 2B Ethidium Bromide red fluorescence and 2C Nomarsky-DIC image.

## References

[b1] Archer GL (1998). *Staphylococcus aureus*: a well-armed pathogen. Clin Infect Dis.

[b2] Aroian R, van der Goot FG (2007). Pore-forming toxins and cellular non-immune defenses (CNIDs). Curr Opin Microbiol.

[b3] Baba Moussa L, Werner S, Colin DA, Mourey L, Pédelacq JD, Samama JP (1999). Discoupling the Ca^2+^-activation from the pore-forming function of the bi-component Panton-Valentine leucocidin in human PMNs. FEBS Lett.

[b4] Batchelor RH, Zhou M (2004). Use of cellular glucose-6-phosphate dehydrogenase for cell quantitation: applications in cytotoxicity and apoptosis assays. Anal Biochem.

[b5] Bird GS, DeHaven WI, Smyth JT, Putney JW (2008). Methods for studying store-operated calcium entry. Methods.

[b6] Brailoiu E, Hoard JL, Filipeanu CM, Brailoiu GC, Dun SL, Patel S, Dun NJ (2005). Nicotinic acid adenine dinucleotide phosphate potentiates neurite outgrowth. J Biol Chem.

[b7] Brickley SG, Farrant M, Swanson GT, Cull-Candy SG (2001). CNQX increases GABA-mediated synaptic transmission in the cerebellum by an AMPA/kainate receptor-independent mechanism. Neuropharmacology.

[b8] Ceni C, Pochon N, Brun V, Muller-Steffner H, Andrieux A, Grunwald D (2003). CD38-dependent ADP-ribosyl cyclase activity in developing and adult mouse brain. Biochem J.

[b9] Chambers HF, DeLeo FR (2009). Waves of resistance: *Staphylococcus aureus* in the antibiotic era. Nat Rev Microbiol.

[b10] Chapman J, Zhou M (1999). Microplate-based fluorometric methods for the enzymatic determination of l-glutamate: application in measuring l-glutamate in food samples. Anal Chim Acta.

[b11] Choorit W, Kaneko J, Muramoto K, Kamio Y (1995). Existence of a new protein component with the same function as the LukF component of leukocidin or [gamma]-hemolysin and its gene in *Staphylococcus aureus* P83. FEBS Lett.

[b12] Churchill GC, Okada Y, Thomas JM, Genazzani AA, Patel S, Galione A (2002). NAADP mobilizes Ca^2+^ from reserve granules, lysosome-related organelles, in sea urchin eggs. Cell.

[b13] Collin T, Marty A, Llano I (2005). Presynaptic calcium stores and synaptic transmission. Curr Opin Neurobiol.

[b14] Collins SR, Meyer T (2011). Evolutionary origins of STIM1 and STIM2 within ancient Ca^2+^ signaling systems. Trends Cell Biol.

[b15] Cosker F, Cheviron N, Yamasaki M, Menteyne A, Lund FE, Moutin M-J (2010). The Ecto-enzyme CD38 is a nicotinic acid adenine dinucleotide phosphate (NAADP) synthase that couples receptor activation to Ca^2+^ mobilization from lysosomes in pancreatic acinar cells. J Biol Chem.

[b16] Crémieux A-C, Dumitrescu O, Lina G, Vallee C, Côté J-F, Muffat-Joly M (2009). Panton-valentine leukocidin enhances the severity of community-associated methicillin-resistant *Staphylococcus aureus* rabbit osteomyelitis. PLoS ONE.

[b18] David MZ, Daum RS (2010). Community-associated methicillin-resistant *Staphylococcus aureus*: epidemiology and clinical consequences of an emerging epidemic. Clin Microbiol Rev.

[b17] David MZ, Boyle-Vavra S, Zychowski DL, Daum RS (2011). Methicillin-susceptible *Staphylococcus aureus* as a predominantly healthcare-associated pathogen: a possible reversal of roles?. PLoS ONE.

[b19] De Bernard M, Cappon A, Pancotto L, Ruggiero P, Rivera J, Del Giudice G, Montecucco C (2005). The *Helicobacter pylori* VacA cytotoxin activates RBL-2H3 cells by inducing cytosolic calcium oscillations. Cell Microbiol.

[b20] DeLeo FR, Otto M, Kreiswirth BN, Chambers HF (2010). Community-associated meticillin-resistant *Staphylococcus aureus*. Lancet.

[b21] Ferrero E, Orciani M, Vacca P, Ortolan E, Crovella S, Titti F (2004). Characterization and phylogenetic epitope mapping of CD38 ADPR cyclase in the cynomolgus macaque. BMC Immunol.

[b22] Finck-Barbançon V, Prévost G, Piémont Y (1991). Improved purification of leukocidin from *Staphylococcus aureus* and toxin distribution among hospital strains. Res Microbiol.

[b23] Fong KP, Pacheco CMF, Otis LL, Baranwal S, Kieba IR, Harrison G (2006). *Actinobacillus actinomycetemcomitans* leukotoxin requires lipid microdomains for target cell cytotoxicity. Cell Microbiol.

[b24] Foran PG, Mohammed N, Lisk GO, Nagwaney S, Lawrence GW, Johnson E (2003). Evaluation of the therapeutic usefulness of botulinum neurotoxin B, C1, E, and F compared with the long lasting type A. Basis for distinct durations of inhibition of exocytosis in central neurons. J Biol Chem.

[b25] Gekara NO, Westphal K, Ma B, Rohde M, Groebe L, Weiss S (2007). The multiple mechanisms of Ca^2+^ signalling by listeriolysin O, the cholesterol-dependent cytolysin of *Listeria monocytogenes*. Cell Microbiol.

[b26] Genestier A-L, Michallet M-C, Prévost G, Bellot G, Chalabreysse L, Peyrol S (2005). *Staphylococcus aureus* Panton-Valentine leukocidin directly targets mitochondria and induces Bax-independent apoptosis of human neutrophils. J Clin Invest.

[b27] Gillet Y, Issartel B, Vanhems P, Fournet J-C, Lina G, Bes M (2002). Association between *Staphylococcus aureus* strains carrying gene for Panton-Valentine leukocidin and highly lethal necrotising pneumonia in young immunocompetent patients. Lancet.

[b28] Gonzalez M, Bischofberger M, Pernot L, van der Goot F, Frêche B (2008). Bacterial pore-forming toxins: the (w)hole story?. Cell Mol Life Sci.

[b29] Graves SF, Kobayashi SD, Braughton KR, Whitney AR, Sturdevant DE, Rasmussen DL (2012). Sublytic concentrations of *Staphylococcus aureus* Panton-Valentine leukocidin alter human PMN gene expression and enhance bactericidal capacity. J Leukoc Biol.

[b30] Gravet A, Colin DA, Keller D, Girardot R, Monteil H, Prévost G (1998). Characterization of a novel structural member, LukE–LukD, of the bi-component staphylococcal leucotoxins family. FEBS Lett.

[b31] Gruszczynska-Biegala J, Pomorski P, Wisniewska MB, Kuznicki J (2011). Differential roles for STIM1 and STIM2 in store-operated calcium entry in rat neurons. PLoS ONE.

[b32] Grynkiewicz G, Poenie M, Tsien RY (1985). A new generation of Ca^2+^ indicators with greatly improved fluorescence properties. J Biol Chem.

[b33] Hara-Yokoyama M, Kimura T, Kaku H, Wakiyama M, Kaitsu Y, Inoue M (2008). Alteration of enzymatic properties of cell-surface antigen CD38 by agonistic anti-CD38 antibodies that prolong B cell survival and induce activation. Int Immunopharmacol.

[b34] Ishikawa J, Ohga K, Yoshino T, Takezawa R, Ichikawa A, Kubota H, Yamada T (2003). A pyrazole derivative, YM-58483, potently inhibits store-operated sustained Ca^2+^ influx and IL-2 production in T lymphocytes. J Immunol.

[b35] Jayasinghe L, Bayley H (2005). The leukocidin pore: evidence for an octamer with four LukF subunits and four LukS subunits alternating around a central axis. Protein Sci.

[b36] Jover E, Gonzalez de Aguilar J-L, Luu B, Lutz-Bucher B (2005). Effect of a cyclohexenonic long-chain fatty alcohol on calcium mobilization. Eur J Pharmacol.

[b37] Kaneko J, Kimura T, Narita S, Tomita T, Yoshiyuki K (1998). Complete nucleotide sequence and molecular characterization of the temperate staphylococcal bacteriophage ΦPVL carrying Panton-Valentine leukocidin genes. Gene.

[b38] Kao C-Y, Los FCO, Huffman DL, Wachi S, Kloft N, Husmann M (2011). Global functional analyses of cellular responses to pore-forming toxins. PLoS Pathog.

[b39] Kloft N, Busch T, Neukirch C, Weis S, Boukhallouk F, Bobkiewicz W (2009). Pore-forming toxins activate MAPK p38 by causing loss of cellular potassium. Biochem Biophys Res Commun.

[b40] Korzeniowski MK, Popovic MA, Szentpetery Z, Varnai P, Stojilkovic SS, Balla T (2009). Dependence of STIM1/Orai1-mediated calcium entry on plasma membrane phosphoinositides. J Biol Chem.

[b41] Koschinski A, Repp H, Ünver B, Dreyer F, Brockmeier D, Valeva A (2006). Why *Escherichia coli* α-hemolysin induces calcium oscillations in mammalian cells – the pore is on its own. FASEB J.

[b42] Lonchamp E, Dupont J-L, Wioland L, Courjaret R, Mbebi-Liegeois C, Jover E (2010). *Clostridium perfringens* epsilon toxin targets granule cells in the mouse cerebellum and stimulates glutamate release. PLoS ONE.

[b43] Lowy FD (1998). *Staphylococcus aureus* infections. N Engl J Med.

[b46] McGuinness L, Bardo SJ, Emptage NJ (2007). The lysosome or lysosome-related organelle may serve as a Ca^2+^ store in the boutons of hippocampal pyramidal cells. Neuropharmacology.

[b44] Malachowa N, Whitney AR, Kobayashi SD, Sturdevant DE, Kennedy AD, Braughton KR (2011). Global changes in *Staphylococcus aureus* gene expression in human blood. PLoS ONE.

[b45] Malavasi F, Deaglio S, Funaro A, Ferrero E, Horenstein AL, Ortolan E (2008). Evolution and function of the ADP ribosyl cyclase/CD38 gene family in physiology and pathology. Physiol Rev.

[b47] Menestrina G, Dalla Serra M, Comai M, Coraiola M, Viero G, Werner S (2003). Ion channels and bacterial infection: the case of β-barrel pore-forming protein toxins of *Staphylococcus aureus*. FEBS Lett.

[b48] Meyer F, Girardot R, Piemont Y, Prevost G, Colin DA (2009). Analysis of the specificity of panton-valentine leucocidin and gamma-hemolysin f component binding. Infect Immun.

[b49] Mhyre TR, Maine DN, Holliday J (2000). Calcium-induced calcium release from intracellular stores is developmentally regulated in primary cultures of cerebellar granule neurons. J Neurobiol.

[b50] Morgan AJ, Platt FM, Lloyd-Evans E, Galione A (2011). Molecular mechanisms of endolysosomal Ca^2+^ signalling in health and disease. Biochem J.

[b51] Pedersen M, Benfield T, Skinhoej P, Jensen A (2006). Haematogenous *Staphylococcus aureus* meningitis. A 10-year nationwide study of 96 consecutive cases. BMC Infect Dis.

[b52] Pinilla PJG, Hernández AT, Camello MC, Pozo MJ, Toescu EC, Camello PJ (2005). Non-stimulated Ca^2+^ leak pathway in cerebellar granule neurones. Biochem Pharmacol.

[b53] Prévost G, Cribier B, Couppié P, Petiau P, Supersac G, Finck-Barbancon V (1995). Panton-Valentine leucocidin and gamma-hemolysin from *Staphylococcus aureus* ATCC 49775 are encoded by distinct genetic loci and have different biological activities. Infect Immun.

[b54] Prévost G, Mourey L, Colin DA, Menestrina G (2001). Staphylococcal poreforming toxins. Curr Top Microbiol Immunol.

[b55] Rahman A, Nariya H, Izaki K, Kato I, Kamio Y (1992). Molecular cloning and nucleotide sequence of leukocidin F-component gene (*lukF*) from methicillin resistant *Staphylococcus aureus*. Biochem Biophys Res Commun.

[b56] Randall A, Tsien RW (1995). Pharmacological dissection of multiple types of Ca^2+^ channel currents in rat cerebellar granule neurons. J Neurosci.

[b57] Sheen T, Ebrahimi C, Hiemstra I, Barlow S, Peschel A, Doran K (2010). Penetration of the blood–brain barrier by *Staphylococcus aureus*: contribution of membrane-anchored lipoteichoic acid. J Mol Med.

[b58] Singhal D, Foreman A, Bardy J-J, Wormald P-J (2011). *Staphylococcus aureus* biofilms. Laryngoscope.

[b59] Staali L, Monteil H, Colin DA (1998). The staphylococcal pore-forming leukotoxins open Ca^2+^ channels in the membrane of human polymorphonuclear neutrophils. J Membr Biol.

[b60] Uhlen P, Laestadius A, Jahnukainen T, Soderblom T, Backhed F, Celsi G (2000). α-Haemolysin of uropathogenic *E. coli* induces Ca^2+^ oscillations in renal epithelial cells. Nature.

[b61] Usmani SM, von Einem J, Frick M, Miklavc P, Mayenburg M, Husmann M (2012). Molecular basis of early epithelial response to streptococcal exotoxin: role of STIM1 and Orai1 proteins. Cell Microbiol.

[b62] Varnai P, Hunyady L, Balla T (2009). STIM and Orai: the long-awaited constituents of store-operated calcium entry. Trends Pharmacol Sci.

[b63] Venkiteswaran G, Hasan G (2009). Intracellular Ca^2+^ signaling and store-operated Ca^2+^ entry are required in Drosophila neurons for flight. Proc Natl Acad Sci USA.

[b64] Walsh CM, Chvanov M, Haynes LP, Petersen OH, Tepikin AV, Burgoyne RD (2009). Role of phosphoinositides in STIM1 dynamics and store-operated calcium entry. Biochem J.

[b65] Werner S, Colin DA, Coraiola M, Menestrina G, Monteil H, Prevost G (2002). Retrieving biological activity from LukF-PV mutants combined with different S components implies compatibility between the stem domains of these staphylococcal bicomponent leucotoxins. Infect Immun.

[b66] Wertheim HFL, Melles DC, Vos MC, van Leeuwen W, van Belkum A, Verbrugh HA, Nouwen JL (2005). The role of nasal carriage in *Staphylococcus aureus* infections. Lancet Infect Dis.

[b67] Woodin AM (1960). Purification of the two components of leucocidin from *Staphylococcus aureus*. Biochem J.

[b68] Woodin AM, Wieneke AA (1963). The accumulation of calcium by the polymorphonuclear leucocyte treated with staphylococcal leucocidin and its significance in extrusion of protein. Biochem J.

[b69] Yamada M, Mizuguchi M, Otsuka N, Ikeda K, Takahashi H (1997). Ultrastructural localization of CD38 immunoreactivity in rat brain. Brain Res.

[b70] Zhu MX, Ma J, Parrington J, Calcraft PJ, Galione A, Evans AM (2009). Calcium signaling via two-pore channels: local or global, that is the question. Am J Physiol Cell Physiol.

[b71] Zivkovic A, Sharif O, Stich K, Doninger B, Biaggio M, Colinge J (2011). TLR 2 and CD14 mediate innate immunity and lung inflammation to staphylococcal Panton-Valentine leukocidin *in vivo*. J Immunol.

[b72] Zocchi E, Usai C, Guida L, Franco L, Bruzzone S, Passalacqua M, De Flora A (1999). Ligand-induced internalization of CD38 results in intracellular Ca^2+^ mobilization: role of NAD^+^ transport across cell membranes. FASEB J.

