# Supplementary figures and images for: Staphylococcal leukotoxins trigger free intracellular Ca2+ rise in neurones, signalling through acidic stores and activation of store-operated channels
Source: Cell Microbiol. 2012 Dec 6;15(5):742–58. doi: 10.1111/cmi.12069 (PMC3654557; doi:10.1111/cmi.12069)

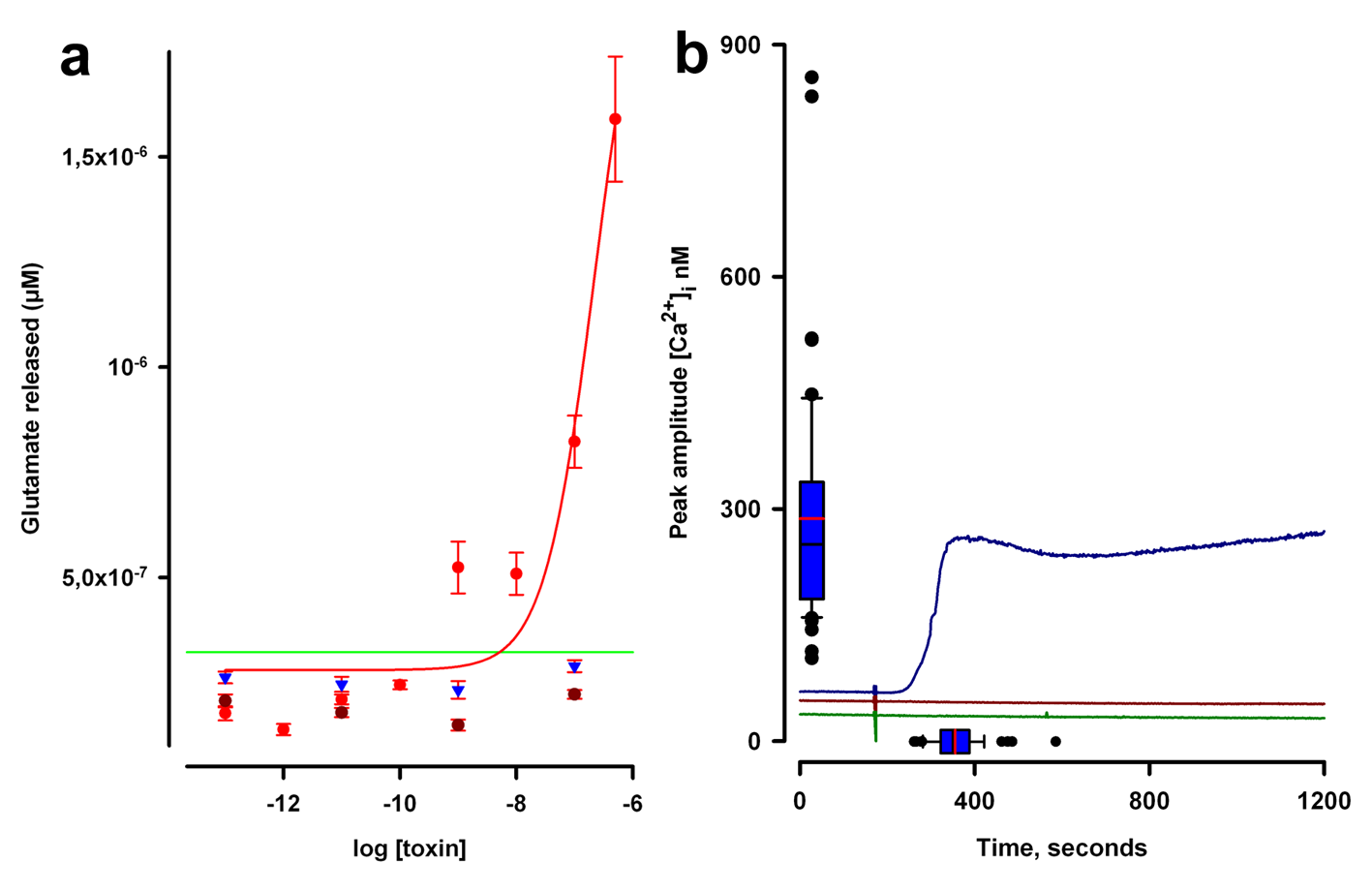

Supplement: Supplementary file 1 [file cmi0015-0742-SD1.zip › cmi_12069_sm_figureS1.tif]

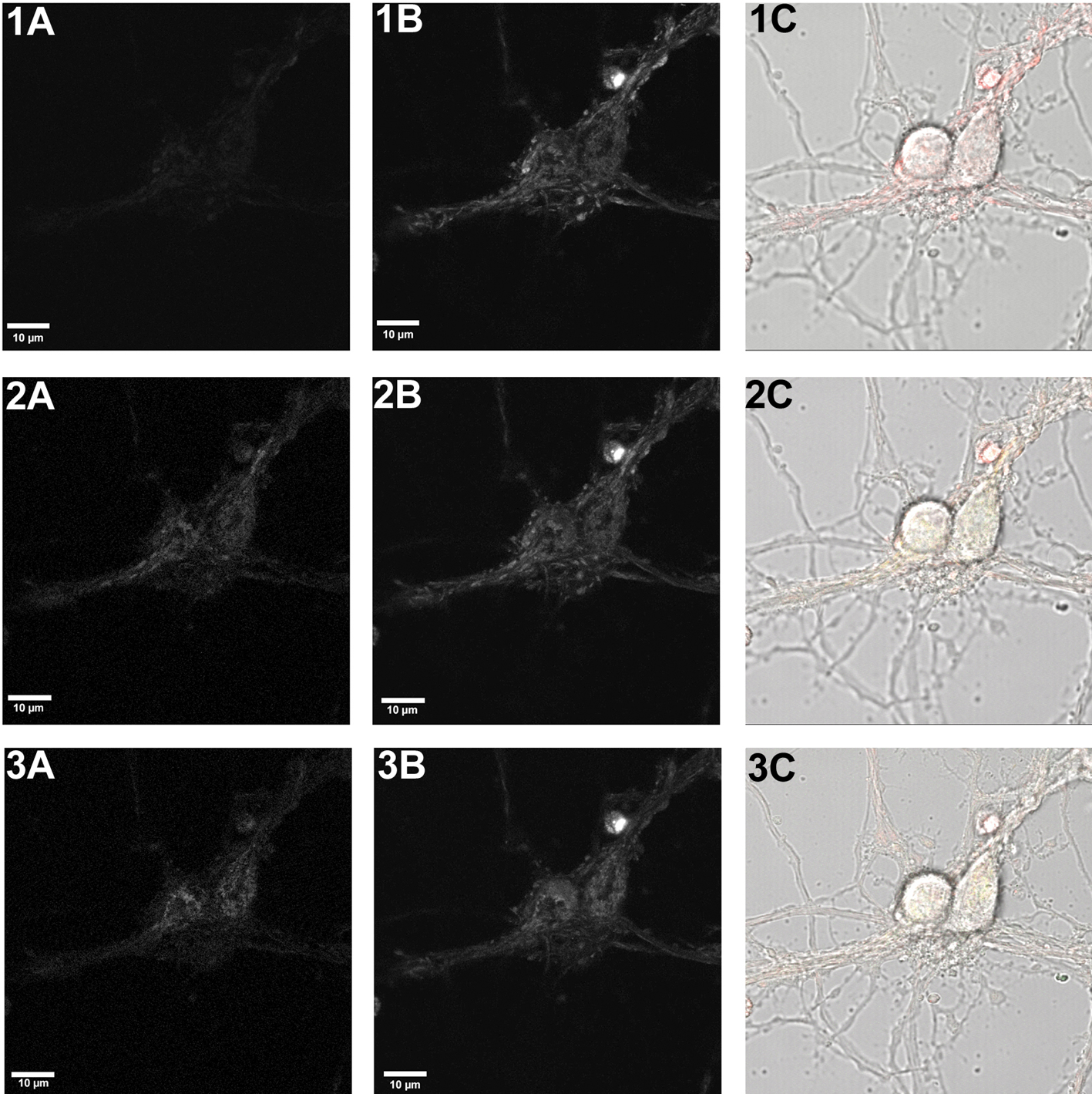

Supplement: Supplementary file 1 [file cmi0015-0742-SD1.zip › cmi_12069_sm_figureS2.tif]
